# Supplementary material for: Stabilization of G-Quadruplexes Modulates the Expression of DNA Damage and Unfolded Protein Response Genes in Canine Lymphoma/Leukemia Cells
Source: Int J Mol Sci. 2025 Oct 12;26(20):9928. doi: 10.3390/ijms26209928 (PMC12563012; doi:10.3390/ijms26209928)
Supplement: Supplementary file 1 [file ijms-26-09928-s001.zip › Supplementary_figures_tables.pdf]

Supplementary figures and tables.

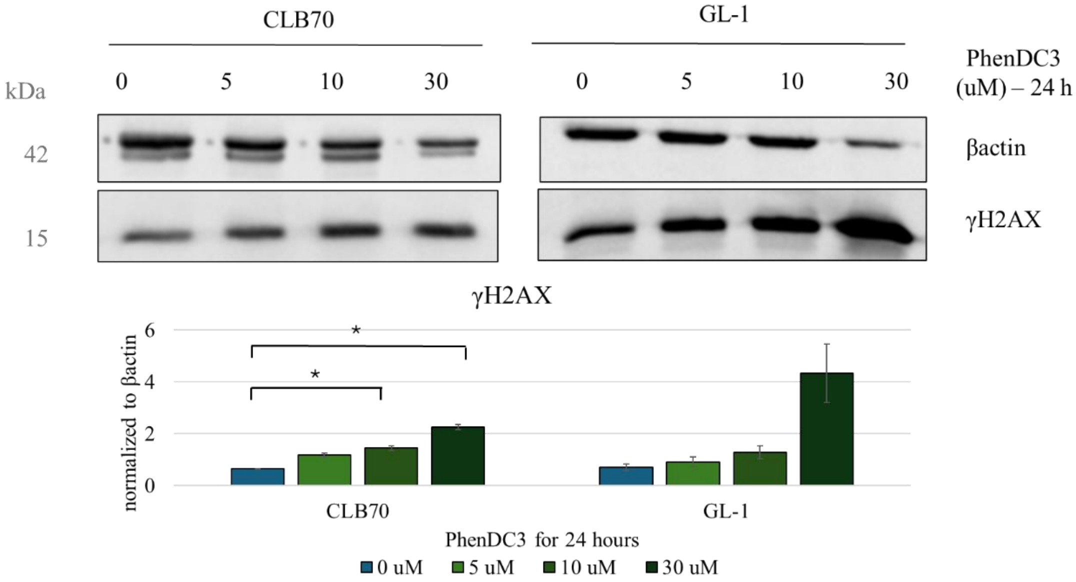

Figure S1. Blot showing the expression of the phosphorylation of H2AX, corresponding with DNA damage on the cell in two selected cell lines without treatment, or treated with PhenDC3 for 24 hours at 5, 10 or 30 µM. A correlation between the increase in the damage and the increase of the concentration of PhenDC3 observed in both cell lines. T-test revealed no significant increase under 5 µM concentration of PhenDC3 in both cell lines. 2 independent experiments measured.

Table S1. RNA quantification and ratios.

Sample ID	[ng/ul]	260/230	260/280
**A_GL1_DMSO**	490,381	2,109	2,091
**A_GL1_PDC3**	377,579	2,136	2,094
**B_GL1_DMSO**	705,039	2,17	2,15
**B_GL1_PDC3**	393,44	2,149	2,111
**C_GL1_DMSO**	193,207	2,153	2,133
**C_GL1_PDC3**	272,62	2,16	2,119
**A_CLB70_DMSO**	694,097	2,14	2,143
**A_CLB70_PDC3**	673,31	2,006	2,139
**B_CLB70_DMSO**	741,477	2,168	2,112
**B_CLB70_PDC3**	732,427	1,925	2,144
**C_CLB70_DMSO**	1155,064	2,035	2,095
**C_CLB70_PDC3**	736,216	2,03	2,118
**A_CLBL1_DMSO**	742,375	2,268	2,001
**A_CLBL1_PDC3**	772,768	1,976	1,937
**B_CLBL1_DMSO**	1287,24	2,142	2,015
**B_CLBL1_PDC3**	1034,598	2,069	2,011
**C_CLBL1_DMSO**	763,423	2,026	2,044
**C_CLBL1_PDC3**	803,392	2,216	2,059

**Samples in Dnase/Rnase free water, total of 20uL*

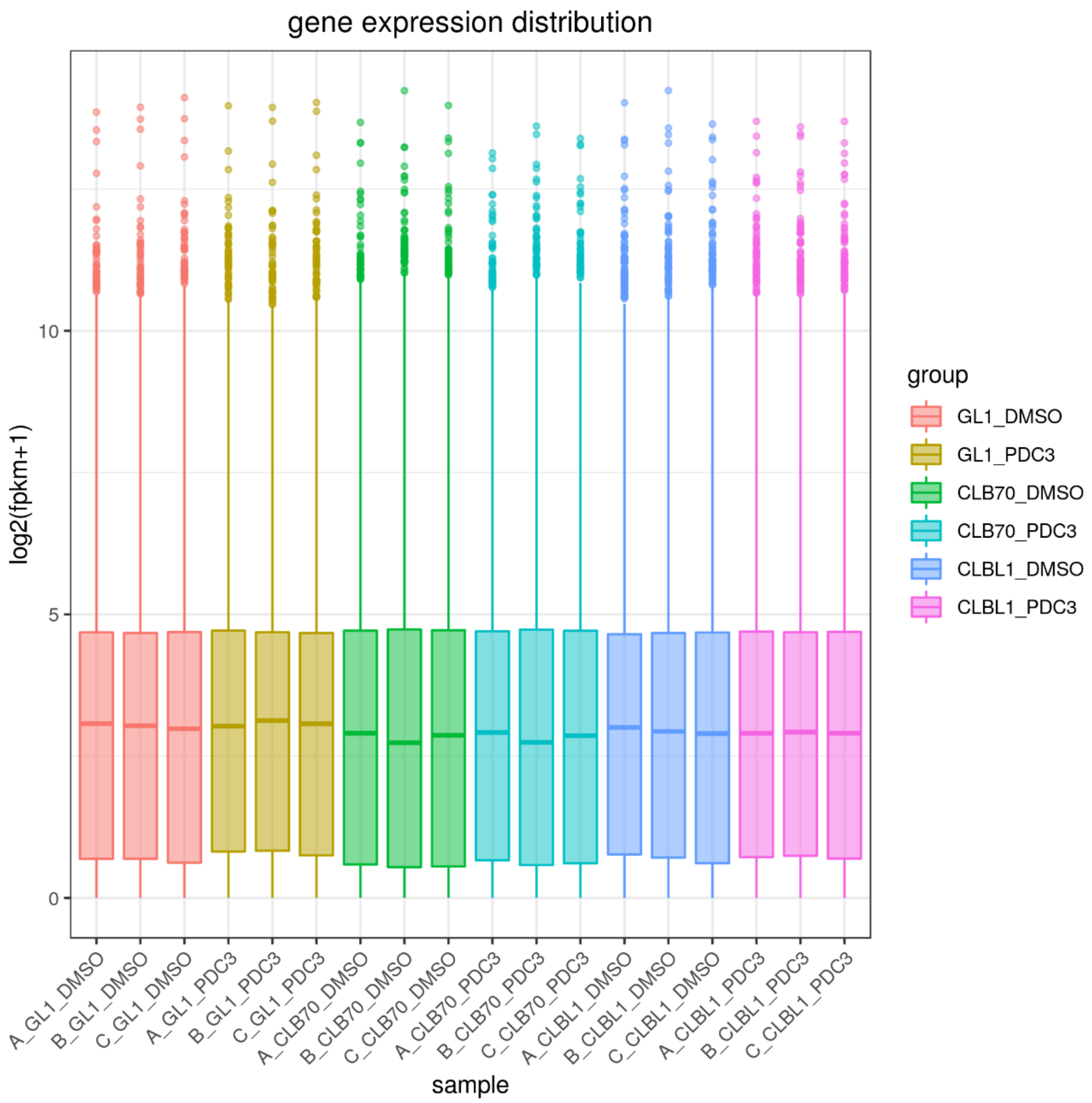

Figure S2. Boxplot representing the distribution of gene expression between the samples analyzed. X axis represents the individual replicates for the 6 biological groups, Y axis indicates the log2(FPKM+1), parameters of box plots are indicated, including maximum, upper quartile, mid-value, lower quartile and minimum. Biological groups are GL-1 DMSO, GL-1 PhenDC3, CLB70 DMSO, CLB70 PhenDC3, CLBL-1 DMSO and CLBL-1 PhenDC3. Three replicates of each: A, B and C. Legend: DMSO – cells treated with DMSO as a control, PDC3 – cells treated with PhenDC3 in a 5 µM concentration for 24 hours. Analysis and figure by Novogene.

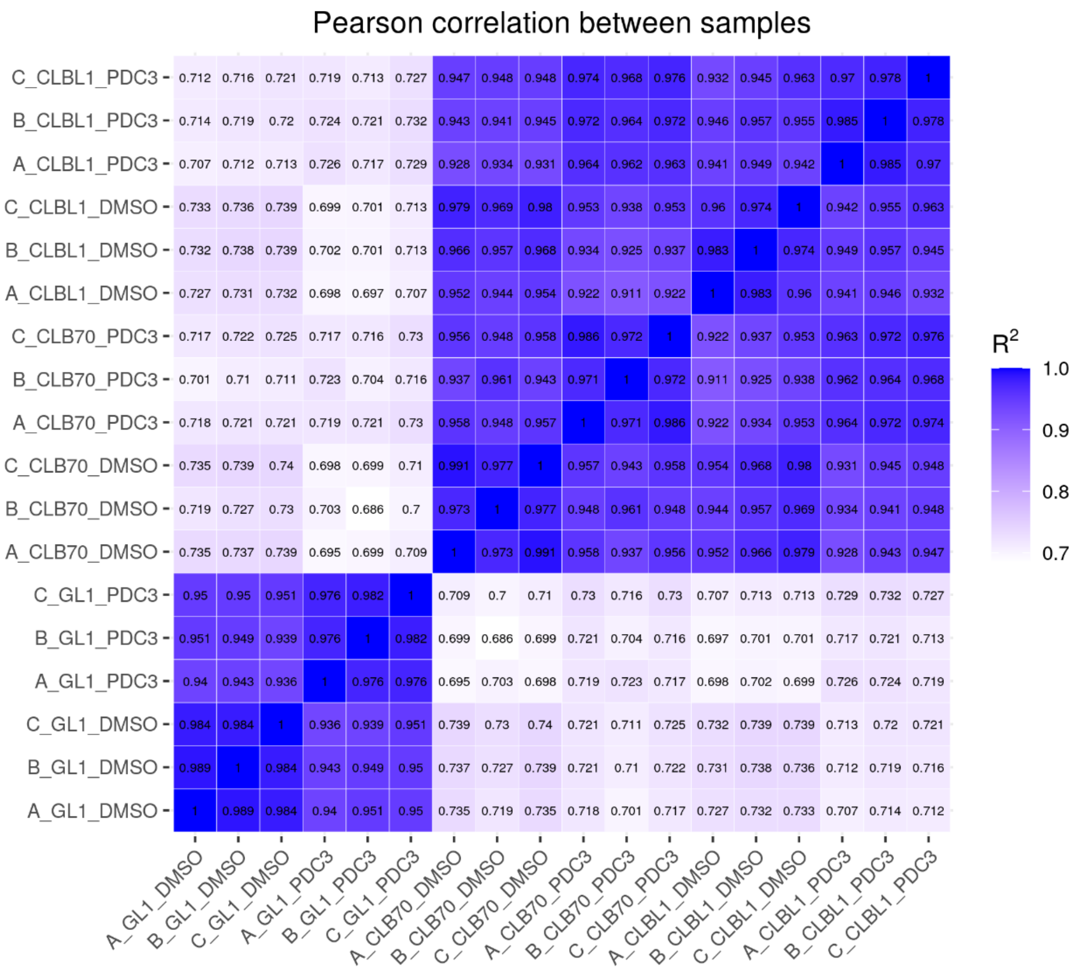

Figure S3. Heat map representing correlation between the different replicate of each biological group. The closer to 1 is the value of the correlation coefficient, the higher is the similarity between the samples. Biological groups are GL-1 DMSO, GL-1 PhenDC3, CLB70 DMSO, CLB70 PhenDC3, CLBL-1 DMSO and CLBL-1 PhenDC3. Three replicates of each: A, B and C. Legend: DMSO – cells treated with DMSO as a control, PDC3 – cells treated with PhenDC3 in a 5 µM concentration for 24 hours. Analysis and figure by Novogene.

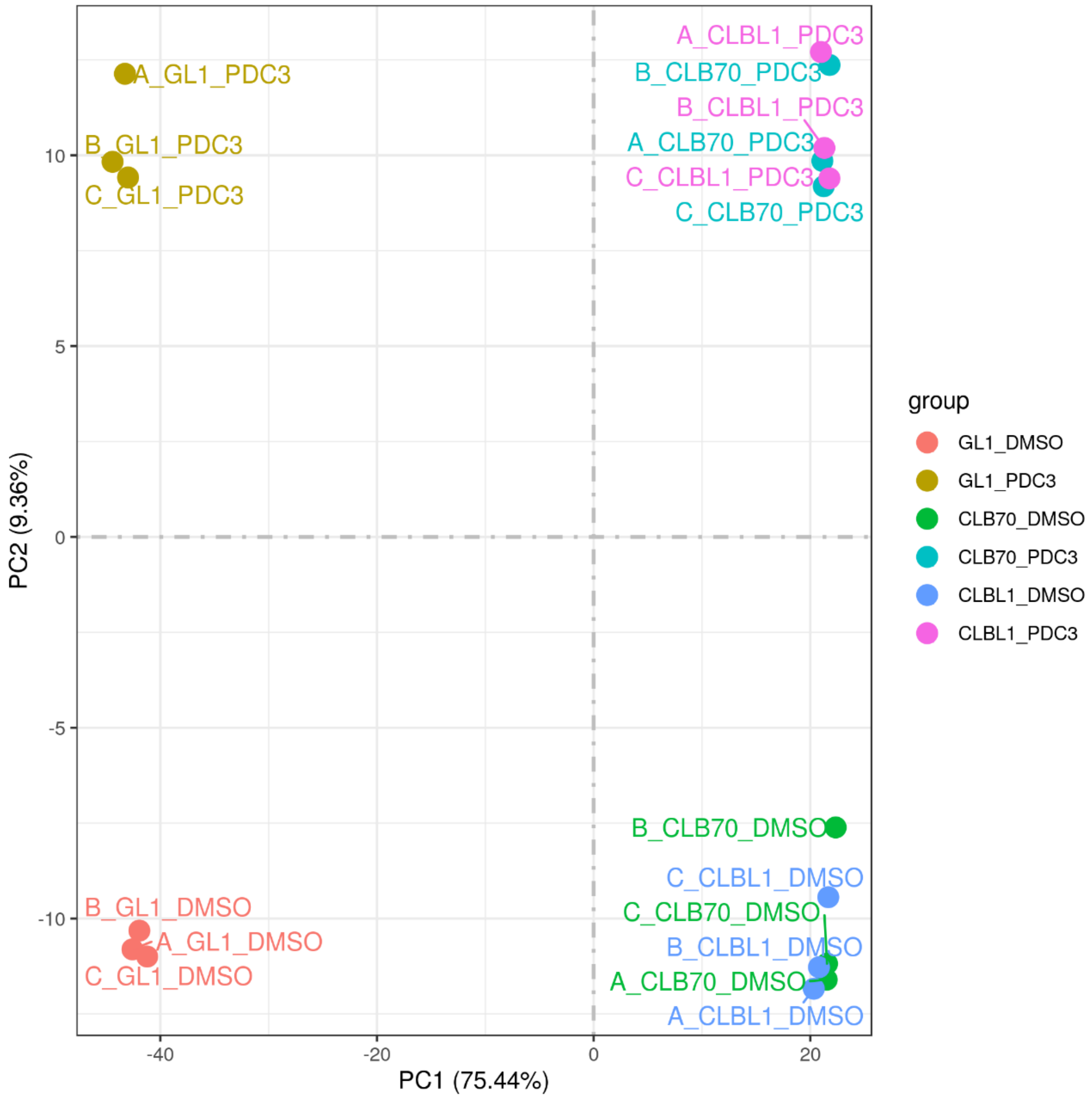

Figure S4. Principal component analysis showing dispersion between the conditions (DMSO and PhenDC3 treatment) and the aggrupation of the three replicates of each condition (A, B, C). Biological groups are GL-1 DMSO, GL-1 PhenDC3, CLB70 DMSO, CLB70 PhenDC3, CLBL-1 DMSO and CLBL-1 PhenDC3. Three replicates of each: A, B and C. Legend: DMSO – cells treated with DMSO as a control, PDC3 – cells treated with PhenDC3 in a 5 µM concentration for 24 hours. Analysis and figure by Novogene.

Table S3. List of genes downregulated and upregulated and log2FoldChange values in the DDR and UPR pathways for the three cell lines tested.

DDR genes					
CLBL-1		CLB70		GL-1	
**gene_name**	**log2FC**	**gene_name**	**log2FC**	**gene_name**	**log2FC**
DVL1	-7,02	*WNT4*	-3,22	*CCNE2*	-1,12
JUN	-5,19	*GADD45A*	-2,62	*RAD51*	-1,73
GADD45A	-4,98	*JUN*	-2,09	*SFN*	-1,47
MDM4	-3,38	*DVL1*	-1,93	*MCM7*	-1,14
PHC3	-2,23	*CBX4*	-1,62	*BCL6*	-1,34
PARP1	-1,91	*TCF7L1*	-1,50	*PIK3R1*	-1,23
AKT1	-1,70	*SFN*	-1,43	*PIK3C2B*	-1,20
CREB1	-1,49	*BCL3*	-1,40	*WNT4*	-1,20
PIK3R1	-1,36	*SNAI1*	-1,19	*FOSL1*	-0,01
WNT4	-1,34	*PIK3R1*	-1,19	*CBX4*	-1,16
PIK3CB	-1,34	*PARP1*	-1,11	*NUP43*	-1,16
SPRED2	-1,14	*PIK3CB*	-1,08	*PSMD14*	-1,46
BCL3	-1,06	*SIRT1*	-1,02	*TFAP4*	-3,14
MYC	-0,21	*PIK3CD*	1,09	*PLK2*	-1,67
SFN	-0,15	*SPRED2*	1,27	*MYC*	1,41
TCF7L1	-0,12	*CCND2*	2,15	*GADD45A*	1,01
CBX4	-0,11			*GADD45B*	1,59
LDLR	1,02			*FOXO3*	1,13
XRCC4	2,00			*PIK3CB*	1,03
				DVL1	1,22
				TCF7L1	1,43
				CCNG2	2,14
				PARP1	1,06
				ANKRD1	1,06

UPR genes					
CLBL-1		CLB70		GL-1	
**gene_name**	**log2FC**	**gene_name**	**log2FC**	**gene_name**	**log2FC**
EDC4	-4,14	*ERP27*	-1,71	*TXNIP*	-3,09
CEBPG	-1,89	*TATDN2*	-1,42	*EXOSC8*	-2,93
CNOT6	-1,78	*CNOT6*	-1,29	*ATF3*	-2,93
PLA2G4B	-1,75	*DDIT4*	-1,29	*ABCA7*	-2,88
TATDN2	-1,62	*NCK2*	-1,27	*PLA2G4B*	-2,33
EIF4EBP1	-1,50	*DNAJB11*	-1,18	*ERP27*	-2,16
SLC7A5	-1,14	*SLC7A5*	-1,17	*EIF4EBP1*	-1,57
NCK2	-0,11	*CEBPG*	-1,08	*NOP56*	-1,31
DNAJB11	0,14	*HERPUD1*	1,02	*TATDN2*	-1,24
		CALR	1,19	*DDIT4*	-1,22
		EIF4EBP1	1,47	*DNAJB9*	-1,20

	FKBP14	2,28	*EXTL1*	-1,15
			GEMIN4	-0,11
			NCK2	1,07
			CCL2	1,25

Table S4. RG4 predicted and experimentally described in human genes. Genes selected based on their downregulation after PhenDC3 treatment on canine cancer cells.

		**Predicted**	**Experimentally**	**Reference**	**Alignment with dog sequence**	
**DDR**	*PARP1*	6	6	[23], [52]	6	2
	PIK3CB	4	-		3	-
	GADD45A	6	1	[23]	6	1
**UPR**	*EIF4EBP1*	3	-		3	-
	NCK2	6	-		5	-
	DDIT4	8	15	[23], [52]	8	15

Predicted RG4 are de novo prediction of RG4s on user-input sequences through three different algorithms. Experimentally RG4 are determined by RG4-Seq or RT-stop-profiling, find in citations. Comparison with dog sequences using BLAST® services [24].

Reference supplementary figures and tables

23. Kwok, C.K.; Marsico, G.; Sahakyan, A.B.; Chambers, V.S.; Balasubramanian, S. RG4-Seq Reveals Widespread Formation of G-Quadruplex Structures in the Human Transcriptome. *Nat. Methods* **2016**, *13*, 841–844. https://doi.org/10.1038/nmeth.3965.
24. Altschul, S. Gapped BLAST and PSI-BLAST: A New Generation of Protein Database Search Programs. *Nucleic Acids Res.* **1997**, *25*, 3389–3402. https://doi.org/10.1093/nar/25.17.3389.
52. Guo, J.U.; Bartel, D.P. RNA G-quadruplexes are globally unfolded in eukaryotic cells and depleted in bacteria. *Science* **2016**, *353*, 6306. https://doi.org/10.1126/science.aaf5371
